# Accurate gingival recession quantification using 3D digital dental models

**DOI:** 10.1007/s00784-022-04795-1

**Published:** 2022-11-23

**Authors:** Konstantinos Dritsas, Demetrios Halazonetis, Mohammed Ghamri, Anton Sculean, Christos Katsaros, Nikolaos Gkantidis

**Affiliations:** 1 Department of Orthodontics and Dentofacial Orthopedics, School of Dental Medicine, University of Bern, Freiburgstrasse 7, CH-3010 Bern, Switzerland; 2 Department of Orthodontics, School of Dentistry, National and Kapodistrian University of Athens, 11527 Athens, Greece; 3 Directorate of Health Affairs-Jeddah, Ministry of Health, Riyadh, Saudi Arabia; 4 Department of Periodontology, School of Dental Medicine, University of Bern, CH-3010 Bern, Switzerland

**Keywords:** Gingival recession, Diagnosis, Digital dentistry, Imaging, Superimposition, Gingival margin

## Abstract

**Objectives:**

To develop and validate a method for accurate quantitative assessment of gingival recessions based on superimposition of serial 3D digital models.

**Materials and methods:**

Gingival recessions of mild (0.5–2 mm) and increased (3–7 mm) severity were simulated on stone casts and surface models were created. The outlines of the gingival margins of the mild (A) and severe recessions (B) were compared to the original gingival margins following 3D best fit superimposition through a gold standard technique (GS), which used intact adjacent structures, and the tested method (CC), which used single tooth crowns at the position of recessions, as superimposition reference. The primary outcome was the distance between the most apical point of each corresponding gingival margin along the respective tooth long axis.

**Results:**

For mild recessions, the median difference of the test methods (CC_A) from the reference method (GS_A) was 0.008 mm (IQR: 0.093; range: − 0.143, 0.147). For severe recessions, the median difference of the test method (CC_B) from the reference method (GS_B) was 0.009 mm (IQR: 0.091; range: − 0.170, 0.198). The proposed method (CC) showed very high intra- and inter-operator reproducibility (median: 0.025 and 0.033 mm, respectively).

**Conclusions:**

The suggested method offers highly accurate monitoring of gingival margin changes and diagnosis of gingival recessions using 3D digital dental models. The method is applicable irrespective of changes in tooth position or form, allowing for assessments over any time span.

**Clinical relevance:**

The accurate detection and visualization of gingival margin changes in 3D will enhance diagnosis and patient-doctor communication.

**Supplementary Information:**

The online version contains supplementary material available at 10.1007/s00784-022-04795-1.

## Introduction

Gingival recessions are becoming increasingly prevalent, as the human life expectancy increases and the access to high-quality dental treatment allows for longer tooth survival [1]. This fact, along with the increased patient demands in regard to oral function and esthetics, designate gingival recession as a problem that modern dentistry is called to manage on a daily basis. At a certain age, every individual will most likely develop gingival recessions due to aging, pathology, inadequate oral hygiene, biomechanical factors, or iatrogenic effects [1]. The exposure of the root surface to the oral environment, occurring when a recession is developed, may cause esthetic concerns, dental hypersensitivity, loss of root structure, loss of tooth vitality, or complete tooth loss, in more extreme cases [2, 3].

The accurate monitoring of changes at gingival margin level over time is crucial for early diagnosis and thorough treatment outcome assessment to facilitate efficient management of current and future patients [2]. In everyday clinical practice, gingival recessions are commonly assessed with a periodontal probe, measuring the distance of the cementoenamel junction (CEJ) to the free gingival margin level. These measurements, however, are operator dependent and can be affected by numerous factors, such as the type of the periodontal probe (width of markings), its orientation, and the necessity to round the values, when the gingival margin falls between two markings [4]. Another drawback of the conventional approach is that the apical shift of the gingival margin can often remain undetected until it surpasses the CEJ, which is difficult to identify if located subgingivally [5].

The aforementioned shortcomings have triggered substantial research over the last few years utilizing the increasing applications of intraoral scanners in modern clinical dentistry. A recent review by Kuralt et al. [6] showcases various methods to assess gingival recessions ex vivo, applied on intraoral photos or 3-dimensional (3D) digital models. Certain studies show promising results, such as higher reproducibility, compared to the conventional method [7, 8]. However, most methods did not take full advantage of the capabilities of 3D imaging, as they reduced the available information to two dimensions, by performing linear measurements of inter-landmark distances either directly on 3D models [7, 8] or on cross-sectional slices [9, 10]. Similar approaches on superimposed 3D models reduce the need of landmark identification by half [11], as they require the identification of two landmarks (only one on each model) instead of four. Any landmark identification induces operator error [12]. Apart from this, the selection of the required landmarks might be arbitrary and does not always reflect the biological rationale of the hypothesis under study [13].

The aim of the present study was to develop and validate an accurate method for 3D visualization and quantification of gingival margin changes, which is based on 3D superimposition of serial digital dental models and would be applicable irrespective of assessment period length or changes in tooth position and form.

## Materials and methods

### Ethical approval

This study was approved by the Research Ethics Committee of the canton of Bern, Switzerland (Project-ID: 2019–00,326, Date: 09/04/2019) and was conducted in accordance with the Helsinki Declaration of 1975, as revised in 2013. All participants signed an informed consent form approving the use of their data for research purposes.

### Sample

Sixteen dental plaster models (type IV plaster, white color, Fujirock EP Premium, GC, Leuven, Belgium) with (*n* = 8; 4 maxillary and 4 mandibular) and without (*n* = 8; 4 maxillary and 4 mandibular) aligned teeth were randomly selected from the archive of the Department of Orthodontics and Dentofacial Orthopedics, University of Bern, Switzerland. The sample consisted of models presenting all teeth in late mixed or permanent dentition, except for third molars, excluding any extreme deviations from a normal morphology (visual assessment by two authors). The crowding on the models without well aligned arches ranged between 4 and 8 mm, whereas on the other models it did not exceed 1 mm. Crowding was measured using the ruler tool of the Viewbox 4 software (version 4.1.0.12 BETA, dHAL Software, Kifissia, Greece, http://www.dhal.com/viewbox.htm), through the sum of the space needed in order to place each malpositioned tooth in a predicted well-aligned dental arch configuration [14].

No formal sample size calculation was performed as the expected/ideal difference between the two compared methods is zero and there are no previous studies that performed similar testing to provide the data required for such a calculation. We selected the sample size empirically as it performed satisfactorily in previous analogous studies on different outcomes [15–17]. Please note that the unit of analysis in the present study is the tooth and not the dental cast. Thus, the actual sample size is 72.

### Gingival recession creation

Gingival recessions were artificially created with manual grinding of the buccal gingival surface of 72 teeth (18 of each tooth type: incisors, canines, premolars, and molars). These teeth were evenly distributed between the maxillary and mandibular models. Various degrees of mild recessions (corresponding to approximately 0.5, 1, and 2 mm of apical shift of the gingival margin) were equally distributed within each tooth type. A pencil mark was placed at a predefined distance apically to the deepest point of the gingival margin of each tooth, using a ruler. A straight handpiece was subsequently used in combination with a laboratory stone knife to grind the gingival surface and simulate the shape of a gingival recession by reaching and removing each pencil mark.

At a second stage, this process was repeated, and each recession was extended apically by additional grinding to simulate recessions of increased severity, corresponding to approximately 3-, 5-, and 7-mm apical shift of the original gingival margin.

The teeth that received simulated recessions were selected in a way that allowed the presence of two intact adjacent teeth within the model. The intact teeth and the adjacent gingival or palatal structures, that were not altered, comprised the stable superimposition reference areas that were used to calculate the gold standard measurements (true value) [15–17].

### Simulation of actual clinical conditions

To simulate actual clinical conditions that occur during normal functioning [18], certain teeth that received artificial recessions were also grinded at the same time at their occlusal surfaces, using a pencil and a laboratory handpiece, as described previously [15, 16]. As a result, various degrees of tooth wear were simulated (corresponding to 0, 1, and 2 mm of vertical tooth loss), which were equally distributed among tooth types and jaws. This step was performed along with the simulation of the mild gingival recessions, thus being present also in the assessment of severe recessions. The actual amount of tooth wear was not measured, as it is beyond the scope of this study.

### 3D model acquisition

The dental casts were scanned at their original status (T0), after mild recession and tooth wear simulation (T1) and once more after increasing the severity of recessions (T2), using a high accuracy 3D surface scanner (stripe light/LED illumination; accuracy < 20 μm; Laboratory scanner D104a, Cendres + Métaux SA, Rue de Boujean 122, CH-2501 Biel/Bienne). The subsequent 3D Standard Tessellation Language (STL) models were then analyzed with Viewbox 4 software.

### Gingival recession measurement workflow

The gingival margin outlines of the mild recessions (T1) were compared to the outlines of the original margins (T0) providing the first set of measurements (A), using the test technique (CC: complete crown) and the gold standard technique (GS). A second set of measurements (B) was obtained by comparing the severe recessions (T2) to the original margins (T0), applying once more both techniques (GS and CC).

**Fig. 1:**
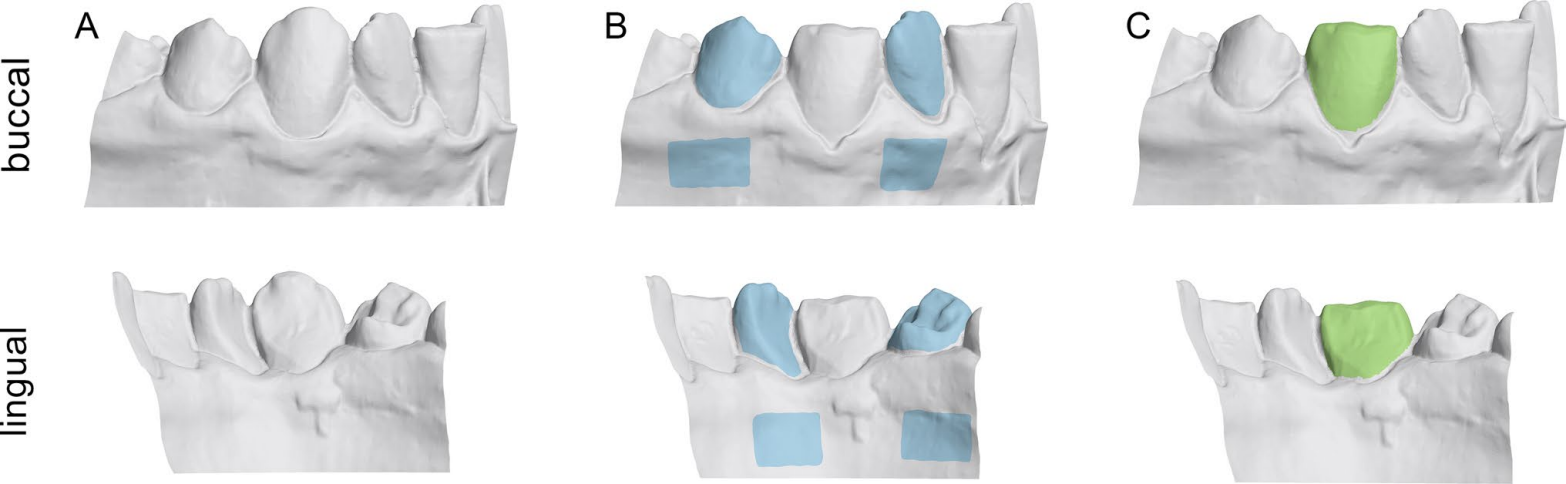
**A** Original surface models. **B** Gold standard reference area (blue). **C** Complete crown reference area (green)

For the gold standard measurements, the adjacent intact teeth and part of the alveolar process were used as superimposition reference area (Fig. 1B). Perfect congruence of the two surface models is expected after a best-fit superimposition of the aforementioned identical surfaces, thus providing optimal spatial relation of the two gingival margins [15–17]. The test method measurements were obtained, by using the complete T1 or T2 clinical crowns as superimposition reference (Fig. 1C). An overview of the variables assessed in this study, with the corresponding abbreviations, is presented in Table 1.

**Table 1:** Overview of the applied superimpositions

Technique	Reference area	Recession severity	Estimated overlap
GS_A	Adjacent intact teeth and alveolar processes	Mild (0.5–2 mm)	100%
CC_A	Complete crown	Mild (0.5–2 mm)	30%
GS_B	Adjacent intact teeth and alveolar processes	Severe (3–7 mm)	100%
CC_B	Complete crown	Severe (3–7 mm)	30%

The superimpositions were performed using the software’s iterative closest point algorithm (ICP) [19], following an initial manual approximation of the 3D objects to assist the automatic registration. Prior to any superimposition and measurement session, the position of the two models was reset to its initial state and the entire measurement process was repeated without reference to previous outcomes. The following superimposition settings were used for the GS measurements: 100% estimated overlap of meshes, matching point to plane, exact nearest neighbor search, exclude overhangs, 100% point sampling, 50 iterations. The CC measurement setting was the same as the GS setting, but with 30% estimated overlap of meshes, based on previous testing [16]. The ICP algorithm was iteratively applied until the distance between the two models was minimized.

**Fig. 2:**
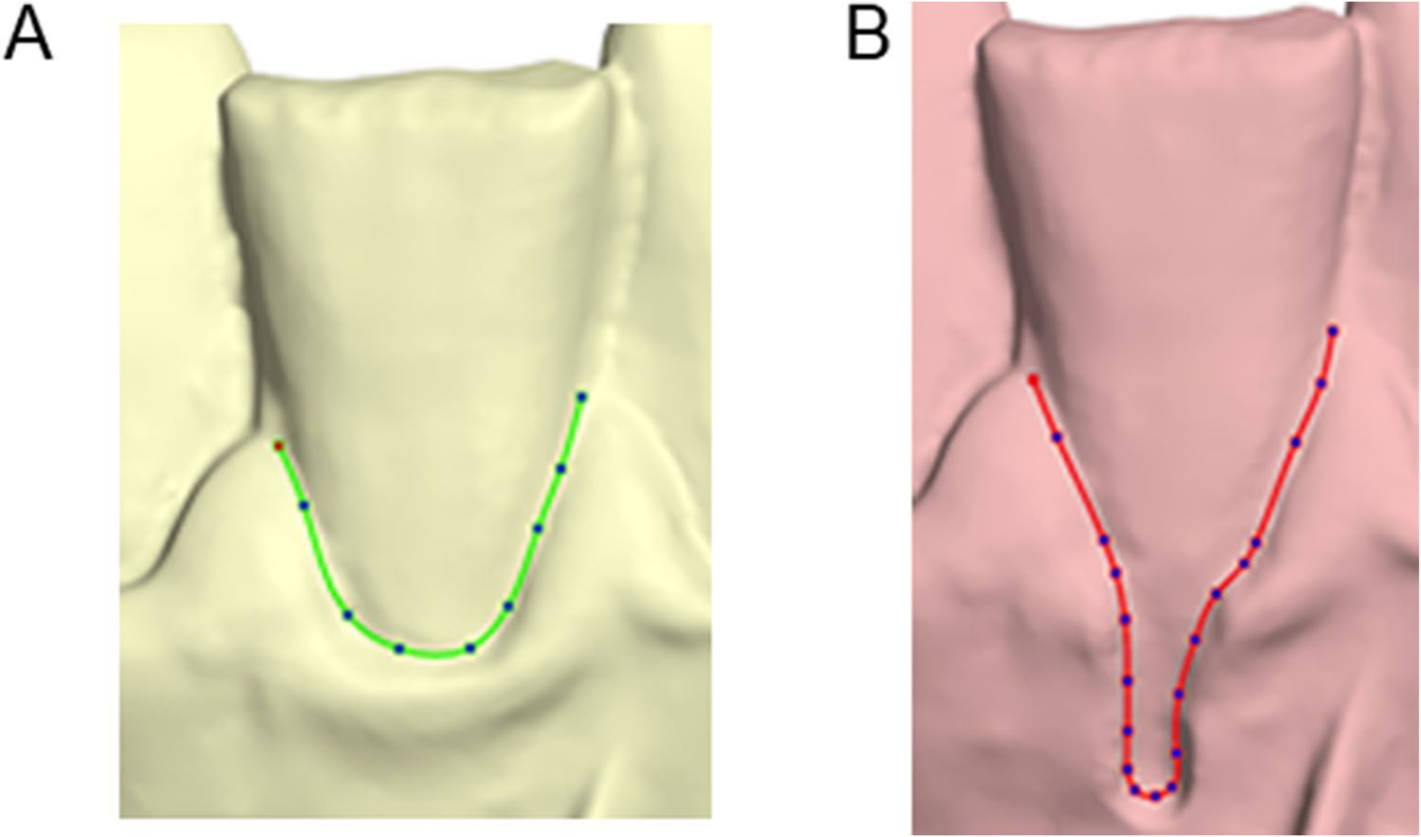
**A** Adjusted curves on the gingival margins at the original model and **B** after the gingival recession

**Fig. 3:**
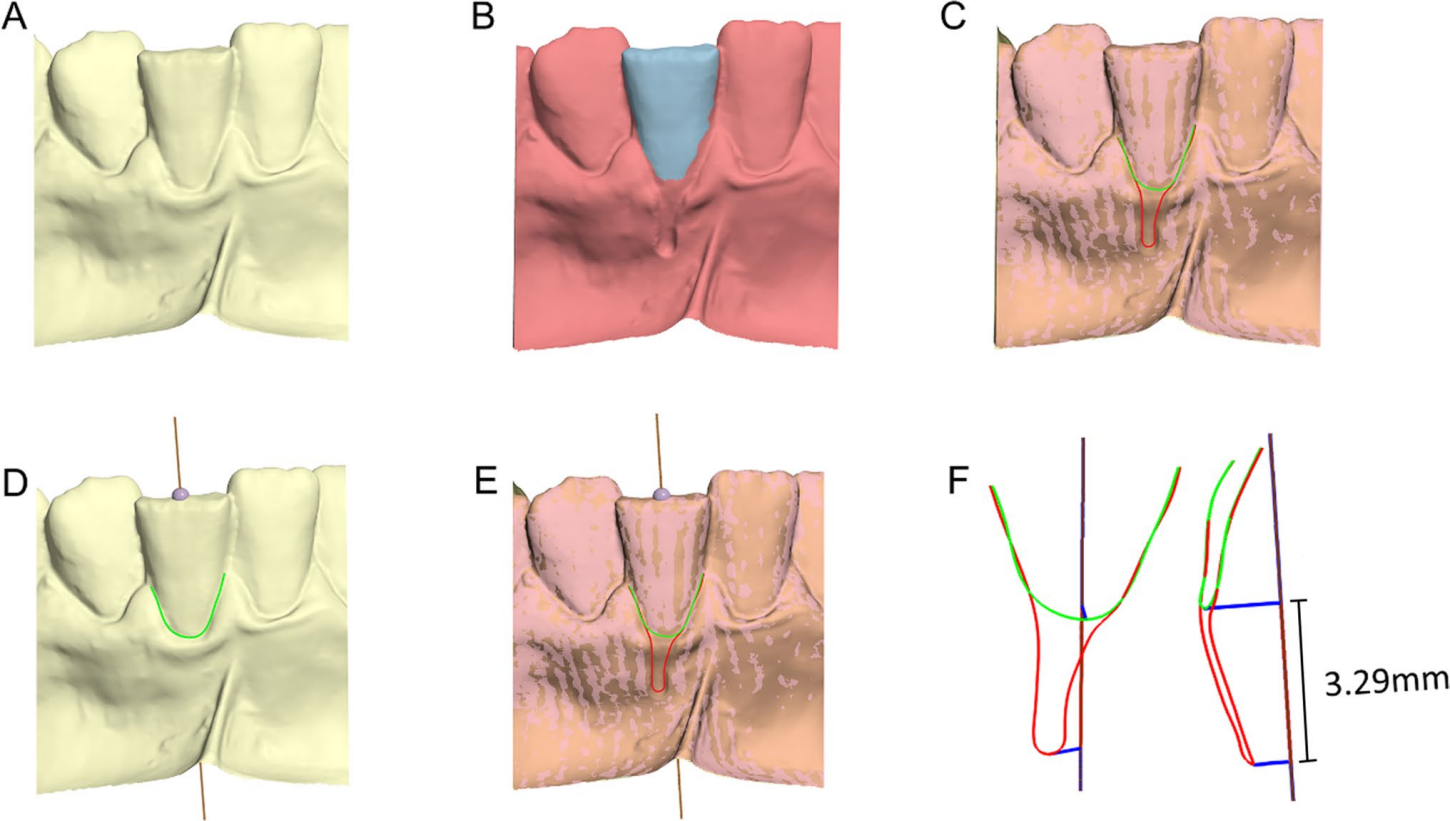
Gingival recession measurement. **A** Original model. **B** Recession model and superimposition reference area (blue). **C** Superimposed models and adjusted curves; colors appear slightly different because of the transparency of the original model. **D** Tooth long axis placement on the original model. **E** Overview of gingival margins and tooth axis. **F** Frontal and lateral view of the adjusted curves and tooth axis configuration; the deepest points of the curves are vertically projected onto the axis (blue lines) and the distance between their projections amounts to the gingival recession

Following each superimposition, the depth of the gingival recession was measured as described below. Firstly, a best-fit occlusal plane was defined by manually positioning landmarks on each tooth cusp or at the middle of the incisal edges of the anterior teeth. This step is required for initial automatic placement of the tooth long axis and the unadjusted preformed gingival margin curves by the software. The preformed curves were manually adjusted to each gingival margin (Fig. 2). Following the curve placement, the tooth long axis was definitely oriented by the operator considering all dimensions of space, through visual assessment of the original tooth model from different viewing angles. The axis passed through the center of each tooth that corresponds to the midpoint of the incisal edge for incisors, the center of the cusp for canines, the midpoint of the central groove for premolars, and the central pit for molars. An automated algorithm was afterwards implemented to vertically project each curve onto the user-defined tooth long axis. Along this axis, the most apical point corresponding to each curve was then automatically selected, and the distance between the two subsequent points was registered as the amount of gingival recession (Fig. 3). The orientation of the axis and the tooth center were reset and redefined before each measurement with the different superimposition techniques.

The amount of gingival recession (mm) that was calculated through the gold standard technique was compared to that of the test technique.

The actual recession measurement process is shown in a Supplementary Video.

### Method error

Intra- and inter-operator error of the test technique (CC) was assessed though the repetition of 32 randomly selected measurements (stratification: 8 measurements per tooth type, split by severity). Intra-operator error of the GS technique was assessed through the repetition of 12 randomly selected measurements (stratification: 3 measurements per tooth type, split by severity).

### Statistical analysis

The statistical analysis was performed by using the IBM SPSS statistics for Windows (Version 28.0. Armonk, NY: IBM Corp). Shapiro–Wilk and Kolmogorov–Smirnov tests were performed on the raw data and certain cases varied from normal distribution. Therefore, non-parametric statistics were applied.

The agreement between the test technique and the gold standard measurement (trueness) in gingival recession assessment was presented in box plots. Zero differences suggest perfect trueness that diminishes the further it deviates from zero. The precision is indicated by the variation of individual values from the median value within each technique. Differences in the precision and trueness between the two techniques and across the severity of the recessions were tested through pairwise comparisons with Wilcoxon’s signed rank test.

The potential effects of tooth type, crowding or tooth wear amount on the precision and trueness of the test technique were tested in an unpaired manner through Kruskal–Wallis tests.

To assess the intra- and inter-operator recession measurement error, overall and for individual assessments, Bland–Altman plots were generated showing the imprecision of each technique as deviation from zero. Systematic differences between groups of repeated measurements were tested through pairwise comparisons using Wilcoxon’s signed rank test.

The significance level was set at an alpha of 0.05. In case of multiple comparisons, a Bonferroni adjustment was applied where needed.

## Results

**Fig. 4:**
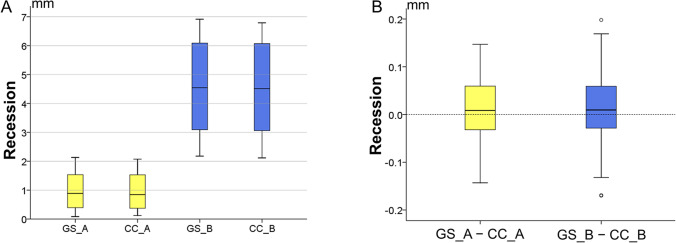
**A** Recession amount measured by the GS and CC techniques. **B** Agreement between GS and CC technique

The complete crown technique (CC) showed perfect agreement with the GS technique. For mild recessions (median: 0.88 mm; IQR: 1.15; range: 0.09, 2.13), the median difference of the test method (CC_A) from the reference method (GS_A) was 0.008 mm (IQR: 0.093; range: – 0.143, 0.147) and was not statistically significantly (*p* = 0.200). For severe recessions (median: 4.54 mm; IQR: 3.02; range: 2.18, 6.92), the median difference of the test method (CC_B) from the reference method (GS_B) was 0.009 mm (IQR: 0.091; range: − 0.170, 0.198) and was also not statistically significant (*p* = 0.121) (Fig. 4).

The difference between the test and reference method for mild recessions (GS_A – CC_A) did not differ significantly from the corresponding difference regarding severe recessions (GS_B – CC_B; *p* = 0.851), indicating that the amount of recession did not affect the reliability of the method.

The trueness of the measurements was not affected by the tooth type, the amount of tooth wear, or the amount of crowding for both mild and severe recessions (*p* > 0.05).

**Fig. 5:**
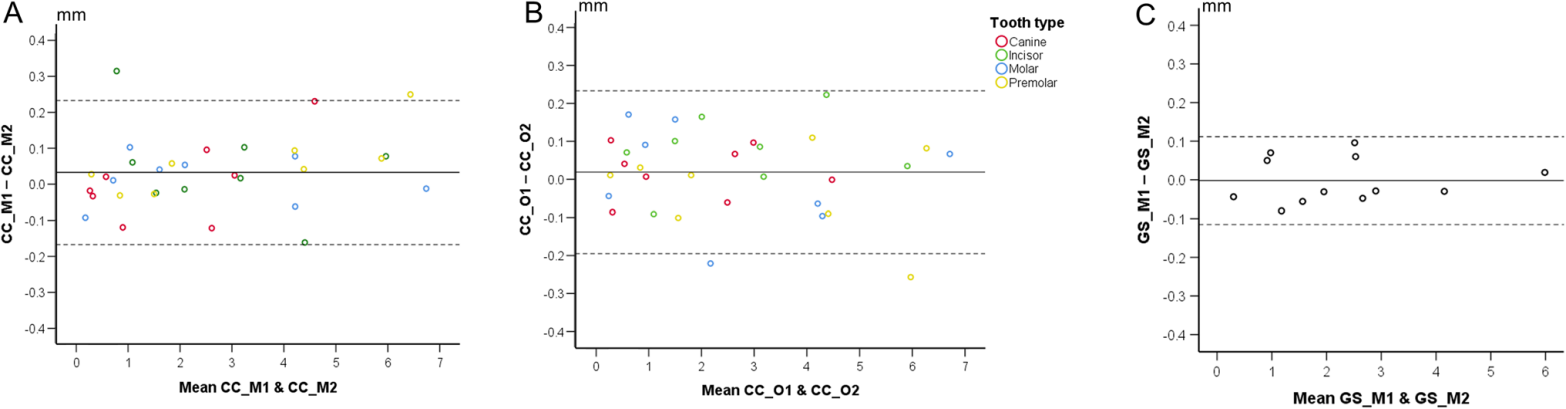
**A** Intra-operator error for the CC technique. **B** Inter-operator error for the CC technique. **C** Intra-operator error for the GS technique

The intra-operator error of the GS technique was negligible in all cases tested (median: − 0.030 mm; IQR: 0.10; range: − 0.08, 0.10) for a median recession of 2.23 mm (IQR:1.81; range: 0.30, 5.99), representing a 1.3% error. No significant difference was found between the repeated measurements (*p* = 1.000). For the CC technique, the median intra-operator error was 0.025 mm (IQR: 0.10; range: − 0.16, 0.31) for a median recession of 2.08 mm (IQR: 3.36; range: 0.17, 6.74), representing a 1.2% error. No significant difference was found between the repeated measurements (*p* = 0.098). The median inter-operator error was 0.033 mm (IQR: 0.16; range: − 0.26, 0.22) for a median recession of 2.09 mm (IQR: 3.41; range: 0.24, 6.71), representing a 1.6% error. No significant difference was found between the repeated measurements (*p* = 0.175). There was no indication that the amount of both inter- and intra-operator error was affected by the amount of recession or by tooth type (Fig. 5).

## Discussion

This study introduces a highly accurate method for the quantitative assessment of gingival margin changes, including gingival recessions, using serial 3D digital dental models. This method essentially consists of two components: the superimposition of the 3D crown surfaces of the assessed tooth, acquired at the two time points of interest, and the calculation of the distance between the gingival margin outlines, measured along the tooth long axis. The complete crown technique (CC) showed perfect agreement with the gold standard (GS) technique, for both mild and severe recessions, indicating that the recession measurement with the CC technique is highly accurate, being at the same time independent of the stability of nearby structures. The result can be visualized in 3D through the software application, which can be a useful tool for the communication between colleagues and enhances doctor-patient communication.

Here, we evaluated recessions on buccal tooth surfaces, but the technique can be similarly applied on lingual surfaces. We also measured differences between the deepest points of each curve, reflecting the current standard of clinical measurements through a periodontal probe. However, the present methodology provides 3D information throughout the entire gingival margin area that can be visualized and quantified at any point of interest. To facilitate this purpose, a plane can be generated passing through the long axis of the tooth of interest. This plane can be freely rotated by the operator, using the tooth long axis as rotation center. For a certain position, the plane will meet the two curves at one point each. The distance of the vertical projections of these points on the tooth long axis will be the gingival margin change at the specific level (Supplementary Fig. 1).

The presented method showed a median trueness of 9 μm considering both mild and severe recessions. To our knowledge, this 3D superimposition study is the first that utilizes a gold standard technique to assess trueness [11, 20]. This is only feasible in an in vitro setting, as there is a lack of stable references when ex vivo methods are used. To simulate various clinical conditions, we considered differences in tooth alignment [21] and introduced tooth wear of different severity among various tooth types [18]. The method proved robust under the quite different circumstances, including morphological or spatial changes in the dentition.

The precision was also quite satisfactory determined at approximately 30 μm. The sources of imprecision include the model superimposition error, the error of the gingival margin curve placement and the error of the tooth long axis placement. Through pilot measurements we deduced that the axis placement had the greatest effect on precision, while the other two components had negligible effects. The error of the single crown superimposition process has been thoroughly tested previously and was minor [15–17, 22]. The gingival margin placement error was also minimal, as revealed by consecutive measurements following repeated curve placements, on previously superimposed crowns, with the tooth long axis held constant. Thus, to enhance accuracy the operator should pay emphasis on proper axis placement in all planes of space, and verify it by viewing the tooth/axis system from various angles.

A recent literature review [6] provided an overview of newly developed 3D methods to assess various characteristics of gingival recessions, such as depth, thickness, area or volume. From the reported studies, only one accessed the recession depth directly using 3D superimpositions [11], whereas the other studies relied on the CEJ as a reference point [7, 23]. Direct methods, such as the one proposed here are able to detect changes in the gingival margin even if the CEJ is not identifiable. At present, despite the high accuracy of the intraoral scanners that reaches 30–50 μm [24], CEJ registration could be problematic due to the presence of saliva or the absence of a tactile surface irregularity (step) [5].

Another major advantage of our method is the possibility to perform a per-tooth analysis. In all previous studies that assessed gingival changes, the entire model or dentition was used as superimposition reference [10, 11, 20]. This may be reliable when assessing very short-term changes but could pose a problem if tooth movement has occurred as a result of normal development, orthodontic treatment, or advanced periodontal disease. In that case, the best fit algorithm will average the changes of individual teeth to achieve an overall best fit, confounding individual measurements [25, 26]. Recent evidence has shown that in certain cases, tooth positional changes can occur even over a time span of 1 week [27]. Using a single tooth crown superimposition technique allows for reliable long-term assessments, irrespective of the final tooth position. Furthermore, the current method is robust in case of incidental tooth wear, which is expected in almost all individuals already at an early age [18]. Lastly, with the present approach any natural or artificial morphological change in one or more teeth of a dentition does not affect the measurements on other teeth.

Various studies have evaluated gingival recessions by superimposing 2D intraoral photographs, which often use a periodontal probe as a reference ruler [11, 28, 29]. Although these methods may be useful in an individual basis for illustration purposes, their accuracy relies heavily on the shooting angle relative to tooth orientation and on the lens distortion effect [30]. Models obtained with intraoral scanners are effectively distortion-free [24, 31].

The proposed method has a wide range of applications in daily practice. Firstly, it can be used to monitor the development of recessions or to detect an apical shift of the gingival margin before the root is exposed. It can also measure gingival margin changes after the correction of tooth inclination through orthodontic treatment [21, 32] or evaluate the outcome of periodontal treatment and hard or soft-tissue augmentation techniques. Furthermore, this method can be a powerful research tool that could shed light to previously inconclusive hypotheses. For example, the evidence on the effect of lower incisor inclination on the development of recessions is contradictory, a fact that was attributed to the lack of standardized, accurate evaluation methods [33–35].

Future research should work to enhance the efficiency and expand the applicability of this method to measure, apart from gingival margin changes, other relevant variables, such as changes in gingival surface area or gingival thickness. Currently, an operator, which has been acclimatized with the software and the superimposition methods, requires approximately 30 min to assess an entire dental arch (14 teeth). A recent study by Kuralt and Fidler [20] illustrated an automated computer-aided gingival margin detection method, based on the increased density of the triangles in areas that present more complex morphology. Modern scanners also offer the possibility to register the coloured texture of the surfaces. This data could also facilitate an automated recognition of the margin, but this remains to be tested. The ultimate goal should be the development of a single software package that automates all steps required to perform an accurate measurement process (single crown superimposition, tooth long axis identification, and gingival margin curve placement).

### Limitations

The technique requires two serial intraoral models of an individual. This might be considered a limitation, compared to single time point assessments, such as direct measurements using a probe. However, serial intraoral models are superior to single clinical assessments as they offer accurate and readily available 3D information on various intraoral structures, including teeth and mucogingival surfaces. Therefore, the acquisition of a 3D digital intraoral model can be seen today as an important adjunction to the periodontal documentation of patients at risk, as well as in orthodontic patients before and after treatment and in research studies on gingival recessions. Ideally, the acquisition of a baseline intraoral model could be performed in every patient at the early permanent dentition as, except for gingival tissue changes, patients can also be precisely monitored for other dental outcomes, including tooth or material wear [15–18] and tooth positional changes [22, 36]. Depending on patient needs, subsequent intraoral models can be obtained on an individually defined time-schedule, facilitating future research and improving clinical decision-making.

Another limitation of this study is that it was performed in vitro to assure a gold standard superimposition reference area, which would not be feasible in a clinical setting. To compensate for this, various clinical conditions were artificially simulated. Furthermore, the used laboratory scanner has higher overall accuracy than the currently available intraoral scanners. However, the error is comparable when small structures, such as single teeth, are considered [24]. Representative sample images are provided in Supplementary Figs. 2 and 3. Lastly, a significant amount of time is needed if this technique is applied in large scale studies, especially when measurements of the entire dentition are required. An experienced user needs about 15 min to measure one dental arch (12 teeth). This time could be shortened through the use of index teeth (e.g., “Ramfjord teeth”) [37] and should not consist a problem for single tooth measurements.

## Conclusions

The present study suggests a single-crown 3D superimposition technique for a highly accurate quantitative assessment of gingival margin changes, including gingival recessions of various severity. The gingival margins can be visualized and evaluated in all dimensions of space. The required 3D surface data can be obtained through readily available intraoral scanners, facilitating relevant research and enabling clinical applications.

## Supplementary Information

Below is the link to the electronic supplementary material.

Supplementary file1 (DOCX 4105 KB)

Supplementary file2 (MP4 15475 KB)

## Data Availability

All data are available in the main text or the extended data. The protocols and datasets generated and/or analysed during the current study are available from the corresponding author on reasonable request.
