# Supplementary material for: Accurate gingival recession quantification using 3D digital dental models
Source: Clin Oral Investig. 2022 Nov 23;27(4):1697–705. doi: 10.1007/s00784-022-04795-1 (PMC10102060; doi:10.1007/s00784-022-04795-1)
Supplement: Supplementary file 1 — Supplementary file1 (DOCX 4105 KB) [file 784_2022_4795_MOESM1_ESM.docx]

**Accurate gingival recession quantification using 3D digital dental models**

Konstantinos Dritsas, Demetrios Halazonetis, Mohammed Ghamri, Anton Sculean, Christos Katsaros, Nikolaos Gkantidis

**
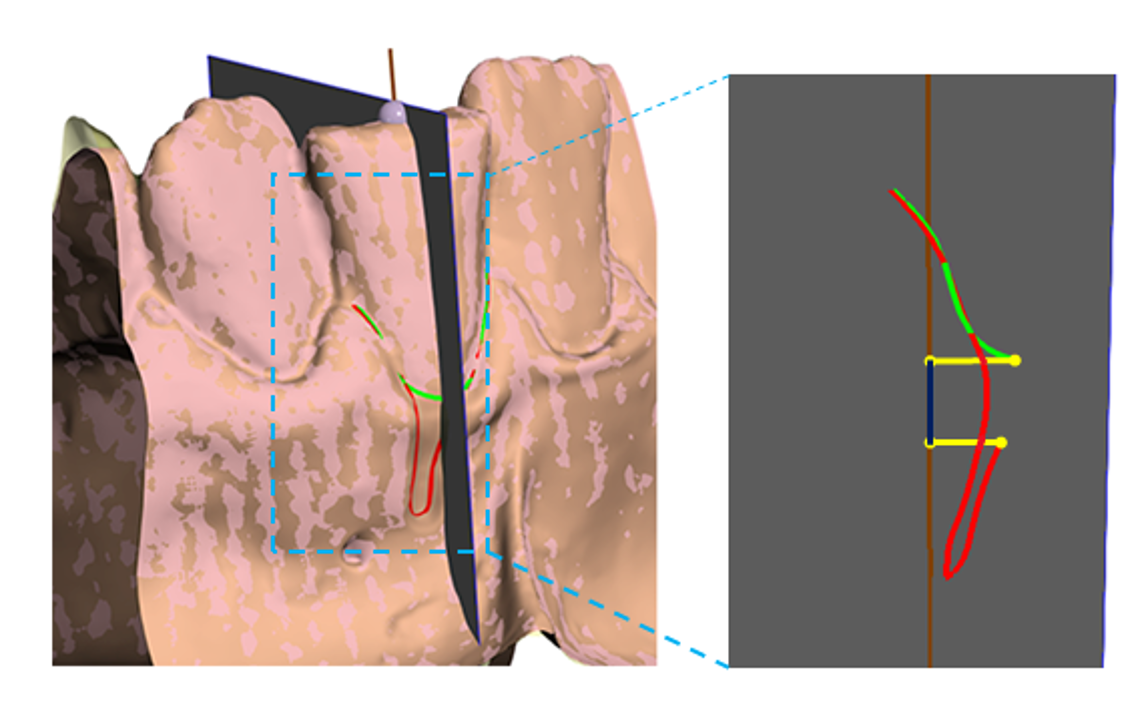
**

**Supplementary Figure 1.** Plane for the assessment of the gingival margin at a certain area. The intersection points of the plane with the gingival margins (yellow points) are projected onto the tooth long axis (yellow lines) resulting to the recession measurement (dark blue line) at the specific level.


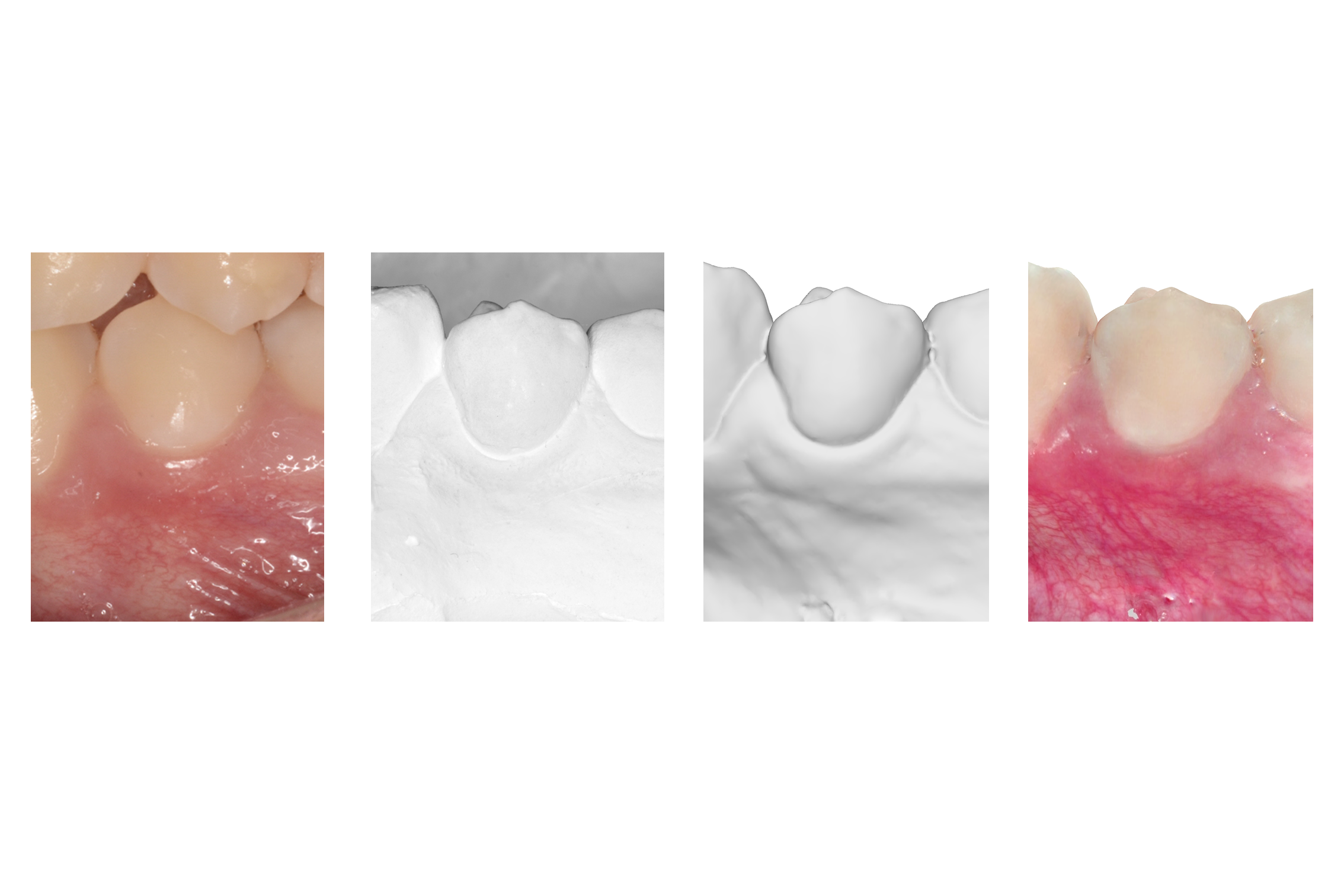


**Supplementary Figure 2.** The gingival margin of a mandibular first premolar as depicted (left to right) on an intraoral photo, a plaster model, an intraoral scan with and without coloured texture (STL and PLY format respectively).


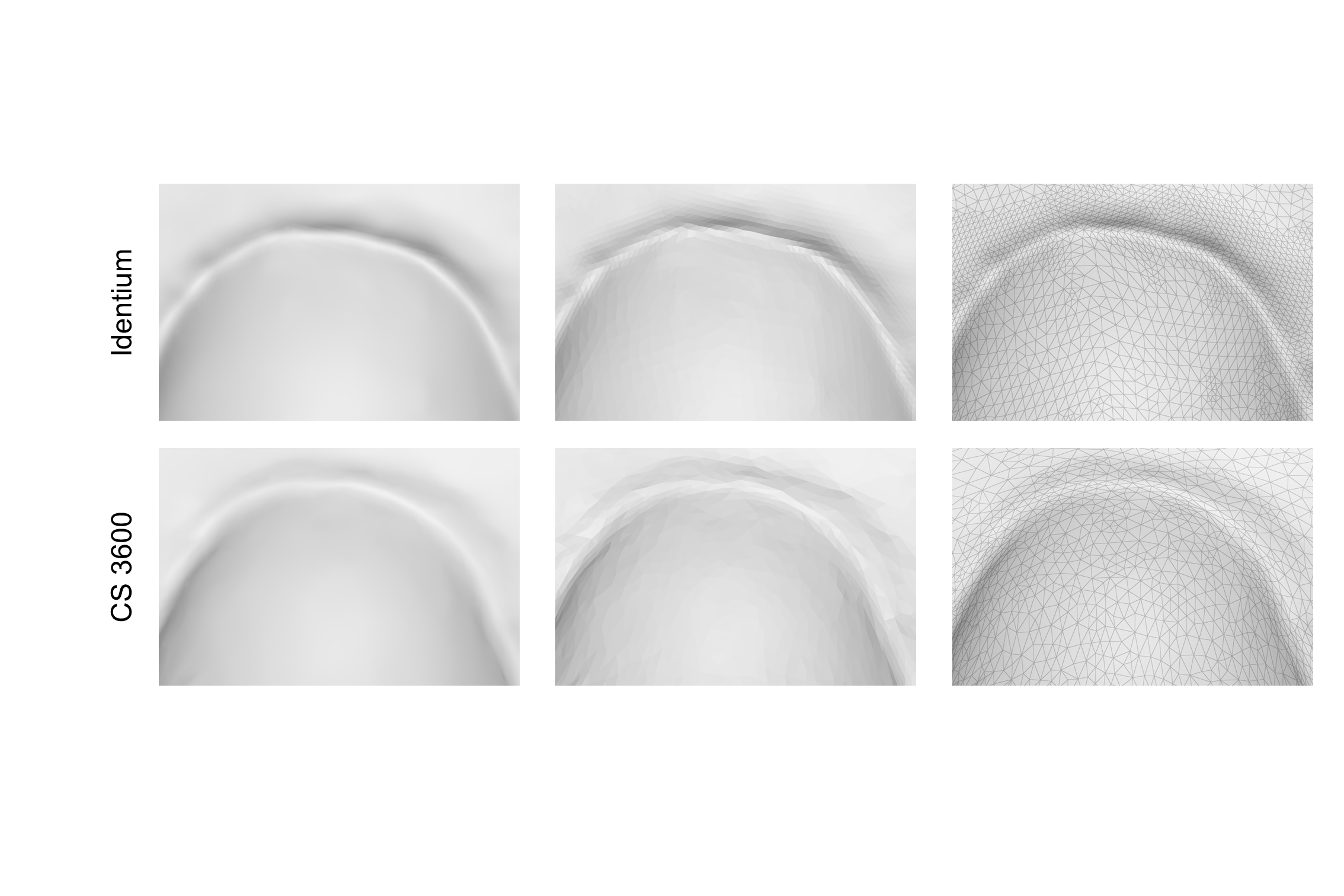


**Supplementary Figure 3.** Different viewing capabilities of the same gingival margin derived from lab scanning (D104a) of a plaster model (Identium impression Kettenbach GmbH & Co. KG, Eschenburg Germany) and from intraoral scanning (CS3600, Carestream, Atlanta USA, Software CS Imaging Version 7.0.23.0.d2). Viewing methods left to right: standard view, faceted view, wireframe view.
